# Free and Cell Wall-Bound Polyamines under Long-Term Water Stress Applied at Different Growth Stages of ×*Triticosecale Wittm*

**DOI:** 10.1371/journal.pone.0135002

**Published:** 2015-08-06

**Authors:** Tomasz Hura, Michał Dziurka, Katarzyna Hura, Agnieszka Ostrowska, Kinga Dziurka

**Affiliations:** 1 The Franciszek Górski Institute of Plant Physiology, Polish Academy of Sciences, 30–239, Kraków, Poland; 2 Department of Plant Physiology, Faculty of Agriculture and Economics, Agricultural University, 30–239, Kraków, Poland; Aberystwyth University, UNITED KINGDOM

## Abstract

**Background:**

Long-stemmed and semi-dwarf cultivars of triticale were exposed to water stress at tillering, heading and anthesis stage. Quantitative determination of free and cell wall-bound polyamines, i.e. agmatine, cadaverine, putrescine, spermidine and spermine, was supplemented with an analysis of quantitative relationships between free and cell wall-bound polyamines.

**Results:**

The content of free and cell wall-bound polyamines varied depending on the development stage, both under optimal and water stress conditions. Drought-induced increase in free agmatine content was observed at all developmental stages in long-stemmed cultivar. A depletion of spermidine and putrescine was also reported in this cultivar, and spermidine was less abundant in semi-dwarf cultivar exposed to drought stress at the three analyzed developmental stages. Changes in the content of the other free polyamines did not follow a steady pattern reflecting the developmental stages. On the contrary, the content of cell wall-bound polyamines gradually increased from tillering, through heading and until anthesis period.

**Conclusion:**

Water stress seemed to induce a progressive decrease in the content of free polyamines and an accumulation of cell wall-bound polyamines.

## Introduction

The role of polyamines in the mechanisms of plant adaptation to various adverse environmental conditions has been widely recognized [1–5]. Some of these studies concern water stress and a positive role of polyamines in leaf dehydration [6–12]. Polyamines are involved in a stabilization of biologically active polyanionic compounds, such as cytoplasmic membrane phospholipides, proteins (including enzymes) or nucleic acids [13,14]. Polyamines modify their activity through interactions of positively charged amino groups and negatively charged groups of macromolecules [15]. It was found that polyamines are directly involved in scavenging reactive oxygen species and can indirectly affect the activity of catalase, peroxidases or superoxide dismutase [16,17]. Other works confirmed a positive effect of polyamines on the activity of the enzymes of Halliwell-Asada pathway that controls the content of non-enzymatic antioxidants such as ascorbate and glutathione [18].

There are also reports indicating direct involvement of polyamines in signal transduction in plants and suggesting a possibility of their indirect effects mediated by interactions with other signal molecules, e.g. nitric oxide or hydrogen peroxide [10,19,20]. Cell wall-bound polyamines allow for maintaining proper thickness of a cell wall and are necessary to maintain cell wall properties by strengthening the bonds between its components [21].

The paragraph above enumerated just the most important functions of polyamines related to their role in plant acclimation to environmental stresses. These compounds have aroused great interest, as on the one hand, the spectrum of their biological effects is very wide, and on the other hand, their action can vary substantially, depending on plant species [22,23]. However, subject literature lacks studies focusing on the changes in polyamine content during key developmental stages in cereals. This is an important issue in terms of searching for reliable defence mechanism under drought stress, as different degree of acclimation to this stress is often reported in the same triticale cultivar depending on its developmental stage. Therefore, the same cultivar can be drought-resistant in the vegetative period, but drought-sensitive at the generative stage [24,25].

The aim of the study was to investigate the changes in the content of free and cell wall-bound polyamines (agmatine, AGM; cadaverine, CAD; putrescine, PUT; spermine, SPM; spermidine, SPD) in triticale plants grown under water stress and in optimal conditions at key stages of their development (tillering, heading, anthesis). This is an innovative field, as such studies have been so far conducted neither in triticale, a synthetic hybrid of wheat and rye, nor in any other cereals.

## Materials and Methods

### Plant material, growth conditions and treatments

The experiments included two cultivars of winter triticale differing in their morphological traits, i.e. 'Moderato', a traditional long-stemmed cultivar and semi-dwarf 'Woltario' cultivar.

Seedlings of each genotype were grown in plastic pots 3.7 dm^3^ in volume, filled with a homogeneous mixture of soil and sand (1:3; v/v). At the stage of one leaf, the seedlings were subjected to vernalization in cool chambers for 8 weeks at +4°C (±1°C), with 10 h illumination and photosynthetic photon flux density (PPFD) of 200 μmol m^-2^ s^-1^. After vernalization, the seedlings were transferred into air-conditioned greenhouse chamber to 16h light/8h dark photoperiod, a temperature of 23/18°C (± 2°C) day/night, 40 ± 5% relative air humidity (RH), and photosynthetic photon flux density (PPFD) of 250–350 μmol m^-2^ s^-1^ (provided by high pressure sodium lamps, 400 W; Philips SON-T AGRO, Brussels, Belgium), at the level of the top leaf. Light intensity at the leaf level was measured with a QSPAR Quantum Sensor (Hansatech Instruments LTD, Kings Lynn, England). The plants were irrigated once a week with a full-strength Hoagland's nutrient solution [26].

In both cultivars, watering was restricted for three weeks at tillering, heading and anthesis, and for two weeks the soil water content (SWC) in drought stress variant was maintained at 34–37% SWC (75–78% SWC for well-watered plants). Soil water content was monitored gravimetrically, taking into account the weight of plants growing in the pots.

Measurements were performed on 21st day of limited watering. Such timing for polyamine content determination was chosen based on our previous experiments on biochemical responses of triticale to water stress [24,25,27–31]. First top fully developed leaves were collected from plants analyzed at tillering stage, whereas the analyses at heading and anthesis involved flag leaves.

### Free and cell wall-bound polyamine extraction

The samples were lyophilized and then pulverized in a mixing mill (MM 400, Retsch, Kroll, Germany). About 15 mg of the sample were weighted and extracted in 1 ml of water solution of formic acid in methanol (4/1/15 v/v) for 15 min at 30 Hz on the mixing mill [32–34]. Then the samples were centrifuged (5 min, 22000 × g, 10°C) and a supernatant was collected. The extraction was repeated and methanolic fraction was pooled. This was the fraction of free phenolics and polyamines (fraction 1). At the next step, the remaining pellet was hydrolyzed with 3M NaOH at 80°C for 15 min and the resulting suspension was neutralized with concentrated hydrochloric acid and the released compounds were extracted as above, yielding the fraction of cell wall-bound polyamines and phenolics (fraction 2). Both fraction (1, 2) extracts were aspirated to dryness under nitrogen stream (TurboVap LV, Capiler Ltd., MA, USA), reconstituted in 2 ml of a sample buffer, i.e. acidified methanolic water solution (4.5% methanol and 4.5% formic acid) and cleaned-up using Discovery DPA-6S SPE cartridges (3 ml, 250 mg, Supelco, Bellefonte, PA, USA). The SPE cartridges were activated with 3 ml of methanol, followed by 3 ml of the sample buffer. After that, the samples were applied, slowly aspirated under vacuum, and cartridges were rinsed with 2 ml of the sample buffer. Both fractions were collected into the same test tube, freeze-dried an kept for polyamine estimation.

### Polyamine dansylation

Biogenic polyamines were estimated according to a modified method described by Marcińska et al. [5]. Dry residue containing polyamine fractions was extracted in 1 ml of 5% HClO_4_ and sonicated for 10 min. Afterwards, the samples were centrifuged at 22000×g for 5 min and the supernatant was collected. Aliquots of the supernatant (0.4 ml) were transferred to 2 ml polypropylene tubes to which 400 μl dansyl chloride solution (5 mg/ml in acetone) and 400 μl saturated sodium carbonate solution were added. The samples were incubated overnight at room temperature. Dansylated polyamines were extracted to 750 μl of toluene in a reaction test tube. The extraction was performed twice and upper toluene layers were collected, combined and evaporated under nitrogen. The dry residue was dissolved in 0.2 ml of methanol, filtered through 0.22 μm membrane and analyzed by HPLC.

### HPLC separation

The HPLC system used was Agilent Infinity 1260 system equipped with a binary pump, an autosampler and a fluorescence detector (FLD). Separation was achieved on Poroshell 120 EC-C18 3.0×50 mm 2.7 μm analytical column (Agilent Technologies), under linear gradient of water with 1% formic acid (A) and methanol (B), from 51% to 80% B in 8 min. Integration time was 10 min. Fluorescence detection was conducted at 350 nm for excitation and 510 nm for emission. Exemplary chromatograms for free and cell wall-bound polyamines in the leaf extract of both winter triticale cultivars are presented in S1 File.

### Statistical analysis

Statistical analysis was carried out using Statistica v. 9.0 (Statsoft Inc.) Analysis of variance was used to determine the main effects of treatments (optimal growth conditions and water stress) and developmental stages (tillering, heading and anthesis) on polyamines content within each studied cultivar. The Duncan's multiple range test at the 0.05 probability level were performed to determine the significance of differences among the treatment means within each cultivar. A replication in an experiment represents a single plant (e.g. 5 replicates means 5 plants).

## Results and Discussion

### Changes in the content of free polyamines

**Fig 1:**
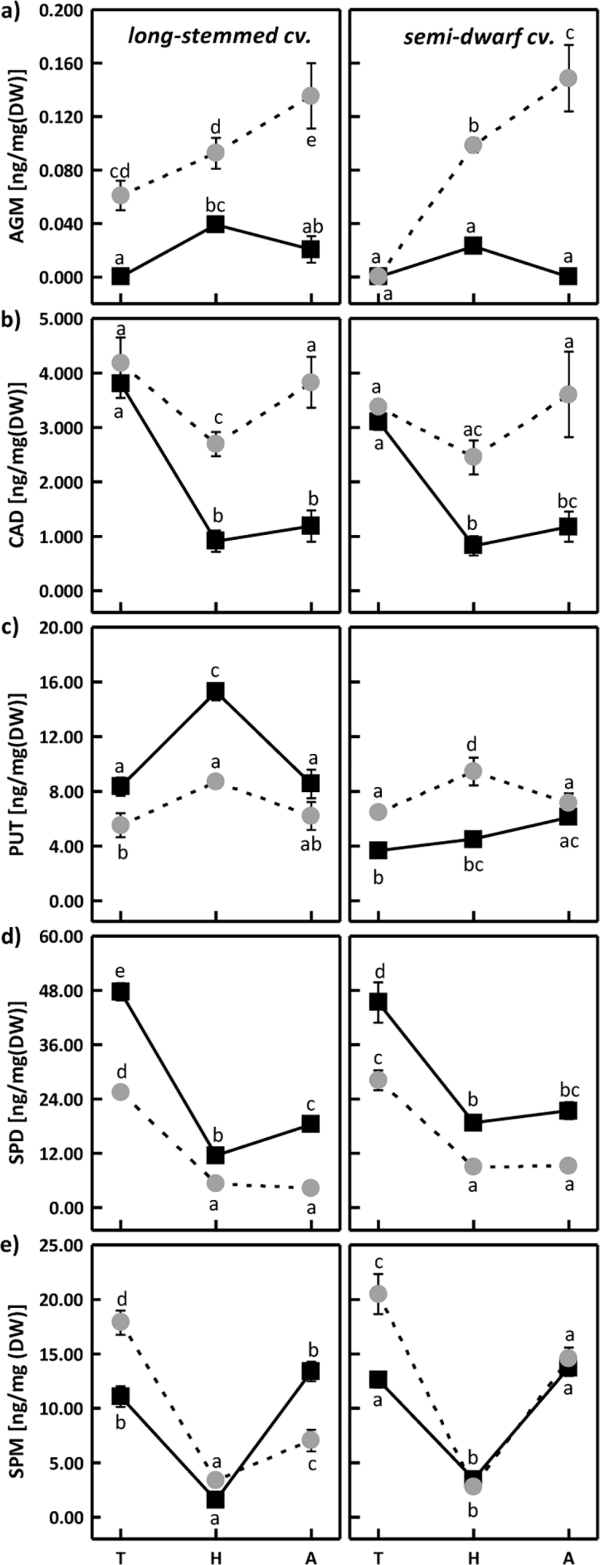
Changes in the content of free agmatine (AGM) (a), cadaverine (CAD) (b), putrescine (PUT) (c), spermidine (SPD) (d) and spermine (SPM) (e) in long-stemmed and semi-dwarf triticale cultivar under optimal growth conditions (black squares) and drought stress (gray circles) at tillering (T), heading (H) and anthesis (A). Means indicated with the same letters within cultivar were not significantly different. Mean values ± SE (n = 5).

Under optimum soil water content, the changes in the level of free agmatine (Fig 1A), cadaverine (Fig 1B), spermidine (Fig 1D), and spermine (Fig 1E) were similar at the same developmental stages in both investigated cultivars.

Significant inter-cultivar differences in the same growing conditions were observed for putrescine (Fig 1C), i.e. its content in long-stemmed cultivar was lower at each developmental stage than in semi-dwarf cultivar. PUT concentration in semi-dwarf cultivar ranged from 3 ng (tillering, heading) to 7 ng (anthesis), while in long-stemmed cultivar it was from 8 ng (tillering, anthesis) to 15 ng per mg dry weight. These differences in the content of free PUT at optimal growth conditions may be due to the fact that PUT is a direct substrate for the synthesis of other polyamines [34], but may also be related to the ontogenesis of long-stemmed and semi-dwarf cultivars, as optimal growth and development are partly controlled by PUT [35]. The highest accumulation of free SPM, SPD and CAD was observed in both cultivars at tillering stage (Fig 1B, 1D and 1E), and of free SPM also during anthesis (Fig 1D and 1E). High content of free PUT was found only in traditional cultivar at heading stage (Fig 1C). These results indicate variable content of polyamines at different developmental stages, which may be associated with their specific functions at individual stages of triticale growth [36]. Polyamines were found to participate in such plant developmental processes as cell divisions, embryogenesis, organogenesis, fruit formation and maturation, leaf senescence, root growth, initiation of anthesis, or tuberization [4,37–39].

In traditional cultivar, a significant increase in AGM accumulation was observed at all developmental stages in the plants exposed to water stress (Fig 1A), while in semi-dwarf cultivar the content of AGM was higher than in control only at heading and anthesis stage. At the same stages, both cultivars of winter triticale showed an elevated level of cadaverine (Fig 1B). Galiba et al. [40] reported an increased accumulation of cadaverine in the calli of sensitive wheat cultivar exposed to mannitol-induced osmotic stress. In another study, Erdei et al. [41] observed higher content of AGM in the leaves of drought-resistant wheat cultivars. It is worth pointing out that AGM concentration under stress conditions may be controlled by the activity of agmatine deiminase converting AGM into PUT, as well as arginine decarboxylase and N-carbamoylputrescine amidohydrolase converting arginine into N-carbamoylputrescine and then into AGM, respectively [42,43].

The pattern of changes was different in the case of free spermidine (Fig 1D), as in both cultivars its level under water stress was lower than in control at all developmental stages. This decline in free SPD could be due to its consumption in SPM biosynthesis and/or oxidation by polyamine oxidase [44]. Marcińska et al. [5] also described a decrease in free SPD in two wheat cultivars during PEG-induced leaf dehydration. Lower than control putrescine level was observed only in traditional cultivar at all analyzed developmental stages (Fig 1C). A similar drop in SPD and PUT content was reported by Nayyar et al. [45], in soybean plants exposed to water stress, which was associated with higher stress injury. In yet another paper, Zhou and Yu [46] reported a decrease in free PUT accompanied by an increase in free SPD in vetiver grass exposed to water stress.

In our study, PUT content in semi-dwarf cultivar under water stress was higher than in control at tillering and heading stage and equal to control during anthesis. Water stress-induced changes in free spermine concentration followed a similar pattern in both cultivars, except for anthesis period (Fig 1E), when SPM content in traditional cultivar was lower than in control, and in semi-dwarf cultivar it was at the control level. In both investigated cultivars of winter triticale, SPM level in plants exposed to drought was higher than in control at tillering stage and equal to control at heading. Increased content of PUT and SPM under water stress, probably resulting from over-expression of arginine decarboxylase (ADC2) and S-adenosylmethionine decarboxylase (SAMDC), respectively, was reported by Alcazár et al. [47] in *A*. *thaliana*. Other papers of this research team [48,49], also revealed a rise in PUT accumulation in transgenic *Arabidopsis*, accompanied by improved resistance to drought stress-induced dehydration. However, a correlation was suggested between increased sensitivity of wheat plants to drought stress and higher concentration of free SPM and SPD [50], which may be also connected to polyamine participation in the scavenging of reactive oxygen species [46].

### Changes in the content of cell wall-bound polyamines

So far, little information is available on the physiological role of cell wall-bound polyamines under water stress. Research papers published on this topic indicate the role of cell wall-bound polyamines in improving hardness and resistance of cell walls to mechanical damage [51,52], morphogenesis [53], or maintaining proper structure of the cell wall [21].

**Fig 2:**
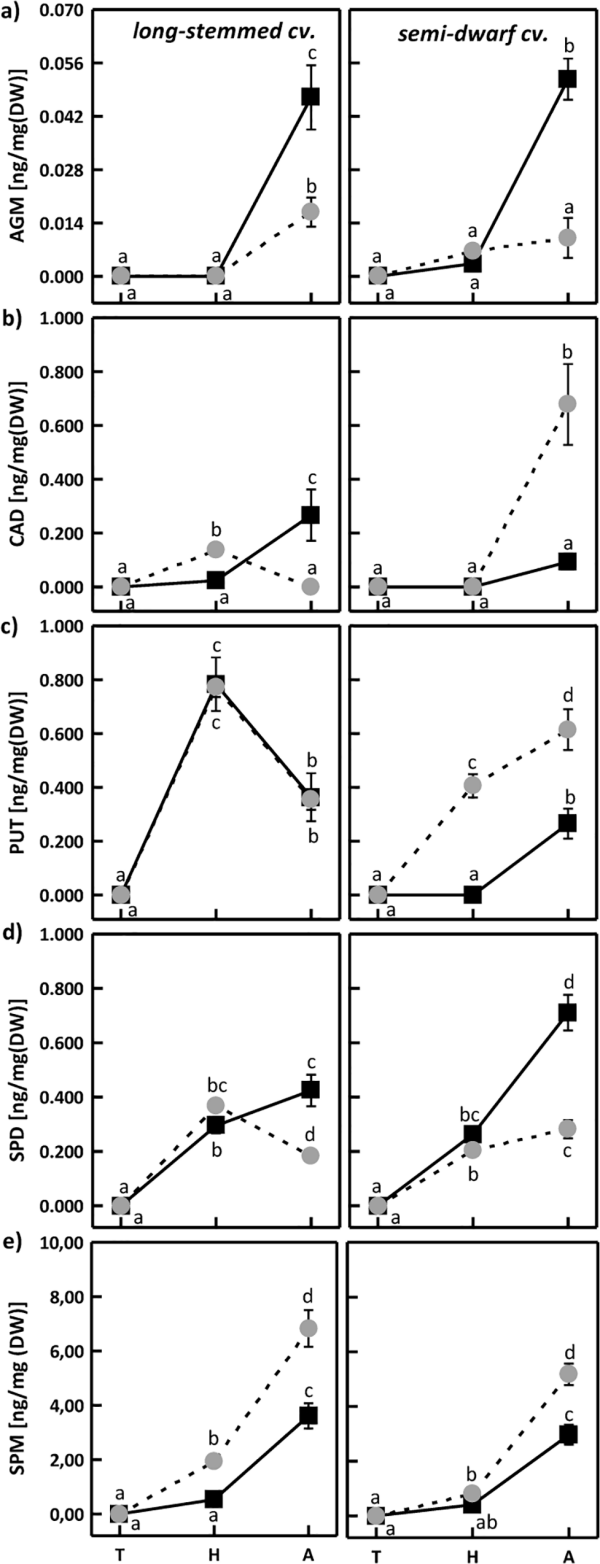
Changes in the content of cell wall-bound agmatine (AGM) (a), cadaverine (CAD) (b), putrescine (PUT) (c), spermidine (SPD) (d) and spermine (SPM) (e) in long-stemmed and semi-dwarf triticale cultivar under optimal growth conditions (black squares) and drought stress (gray circles) at tillering (T), heading (H) and anthesis (A). Means indicated with the same letters within cultivar were not significantly different. Mean values ± SE (n = 5).

Cell wall-bound polyamines were not detected in the investigated cultivars at tillering stage, either in optimal conditions or under water stress (Fig 2).

The highest concentration of cell wall-bound AGM was observed in both cultivars at anthesis and for optimal soil water content, and it was higher than that determined for drought-stressed plants (Fig 2A). At tillering, no cell wall-bound AGM was detected in traditional cultivar, while in semi-dwarf cultivar no clear differences between the experimental variants were found. The presence of AGM linked to cell wall components was confirmed e.g. in the leaves of *Vitis vinifera* [54].

In traditional cultivar, the content of cell wall-bound CAD at heading was significantly higher in drought-exposed plants than in control (Fig 2B). At anthesis, the content of cell wall-bound CAD in the same cultivar and in the same conditions was lower than in control. In contrast, drought stress induced a significant increase in the cell wall-bound CAD in semi-dwarf cultivar at anthesis (Fig 2B). High ability of CAD to conjugate with phenolic compounds was described by Legaz et al. [55]. Polyamines were also reported to function as an anchor binding other molecules, such as phenols, with cell-wall components [21]. Therefore, an increase in cell wall-bound CAD and further binding of e.g. phenolic compounds may strengthen the cell wall. Drought-induced accumulation of cell wall-bound phenolics was already observed in triticale [24,25].

No differences in cell wall-bound PUT content between treatments were found in traditional triticale cultivar at individual developmental stages (Fig 2C). The highest accumulation of cell wall-bound PUT was detected in long-stemmed cultivar at heading, with about 50% reduction at anthesis. In drought-exposed semi-dwarf cultivar, the content of cell wall-bound PUT at heading and anthesis was markedly higher than in control (Fig 2C). Similar pattern regarding the content of cell wall-bound SPD was noticed in the investigated cultivars, both between treatments and within developmental stages (Fig 2D). In both cultivars exposed to water stress, the accumulation of cell wall-bound SPD was the same as in control at heading and lower than in control during anthesis. Drought stress induced an increase in cell wall-bound SPM at heading and anthesis in traditional cultivar, and only at anthesis in semi-dwarf cultivar (Fig 2E). Cell wall-bound polyamines were reported to mediate the formation of bonds between individual cell wall components and between the cell wall and cell membrane [15,56].

### Total content of polyamines and free to cell wall-bound polyamine ratio

Table 1 shows total content of polyamines for specific developmental stages, obtained by summing up mean concentrations of individual polyamines. In both cultivars, a decline in total content of free polyamines, as compared to control, was observed at all developmental stages. An opposite trend, i.e. drought-induced increase, was observed for total content of cell wall-bound polyamines.

**Table 1:** Total content of polyamines (F—free; CW—cell wall-bound) obtained by summing up mean concentration of individual polyamines in long-stemmed and semi-dwarf triticale cultivars under optimal growth conditions (C; control) and water stress (WS) at tillering (T), heading (H) and anthesis (A).

		Long-stemmed cv.			Semi-dwarf cv.		
		T	H	A	T	H	A
**F**	C	67.0 ± 2.89e	29.0 ± 1.78b	41.3 ± 2.36c	61.5 ± 3.31d	27.5 ± 1.64ab	39.5 ± 4.01c
	WS	53.0 ± 2.39d	20.2 ± 0.73a	20.5 ± 2.62a	53.7 ± 5.68d	23.3 ± 2.12a	34.7 ± 3.37bc
**CW**	C	0.0 ± 0.0a	1.09 ± 0.29a	4.60 ± 0.57c	0.0 ± 0.0a	0.67 ± 0.04a	3.72 ± 0.43b
	WS	0.0 ± 0.0a	3.23 ± 0.10b	7.38 ± 0.67d	0.0 ± 0.0a	1.22 ± 0.23a	5.45 ± 1.02c

Means indicated with the same letters within cultivar were not significantly different. Mean values ± SE (n = 5).

A drop in free to cell wall-bound polyamine (F/CW) ratio was usually perceived in plants under water stress, as a consequence of enhanced content of cell wall-bound polyamines in comparison to free polyamines (Table 2). A paper on maturation of mung-bean hypocotyl cells revealed that the content of cell wall-bound polyamines increased in the older hypocotyl cells and was associated with a decline in free polyamines [57]. In traditional cultivar, an increase in F/CW ratio was observed only for AGM during anthesis of drought-exposed plants. In semi-dwarf cultivar, the same phenomenon occurred for AGM and PUT at tillering and AGM and SPD at anthesis.

**Table 2:** The ratio of free to cell wall-bound agmatine (AGM) (a), cadaverine (CAD) (b), putrescine (PUT) (c), spermidine (SPD) (d) and spermine (SPM) (e) in long-stemmed and semi-dwarf triticale cultivar under optimal growth conditions (C; control) and water stress (WS) at tillering (T), heading (H) and anthesis (A).

		Long-stemmed cv.			Semi-dwarf cv.		
		T	H	A	T	H	A
**AGM**	C	-	-	0.4	-	7.7	0.0
	WS	-	-	7.9	-	14.0	14.9
**CAD**	C	-	37.9	4.5	-	-	12.6
	WS	-	19.6	-	-	-	5.3
**PUT**	C	-	19.5	23.5	-	-	23.0
	WS	-	11.3	17.5	-	23.4	11.6
**SPD**	C	-	39.1	43.3	-	71.4	30.2
	WS	-	14.4	22.7	-	44.9	32.8
**SPM**	C	-	2.9	3.7	-	8.6	4.6
	WS	-	1.7	1.0	-	3.4	2.8

Considering huge variability of polyamine content between plant species, individual plants within species and plant organs, Tomar et al. [58] suggested that some polyamines, e.g. cadaverine, could be considered as taxonomic markers. A comparison of heading and anthesis stage performed in our study revealed an increase in cell wall-bound polyamines in relation to free polyamines during anthesis, which was particularly clear in semi-dwarf cultivar both under control and water stress conditions (Table 2).

## Conclusions

The content of free and cell wall-bound polyamines varied in optimal and water stress conditions, depending on the developmental stage of the investigated triticale cultivars. Changes in the content of free polyamines did not follow a steady pattern reflecting the developmental stages. On the contrary, the content of cell wall-bound polyamines gradually increased from tillering, through heading and until anthesis period. Drought stress and successive developmental stages were associated with gradual decline in free and increase in cell wall-bound polyamines. This could be due to the role of polyamines in cell wall strengthening under environmental stress conditions. Growing content of cell wall-bound polyamines during development of semi-dwarf cultivar and an opposite trend observed in long-stemmed cultivar could be specific for the investigated triticale types. However, these results should be verified in similar experiments involving a higher number of semi-dwarf and long-stemmed cultivars. As polyamine contents may vary between and within species, and depend on plant organ and developmental stage, our results may be useful in designing experiments aimed at elucidating the role of specific polyamines in biochemical and molecular mechanisms determining triticale resistance to water stress. Further approaches are required to evaluate three classes of polyamines (free, conjugated, bound) in cell fractions (nuclear, cytosol, membrane, cell wall) to correlate polyamine content with susceptibility to drought stress applied at different growth stages of triticale.

## Supporting Information

S1 File Exemplary chromatograms for free and cell wall-bound polyamines in the leaf extract of both winter triticale cultivars. (DOC)
