# Supplementary material for: Free and Cell Wall-Bound Polyamines under Long-Term Water Stress Applied at Different Growth Stages of ×Triticosecale Wittm
Source: PLoS One. 2015 Aug 6;10(8):e0135002. doi: 10.1371/journal.pone.0135002 (PMC4527768; doi:10.1371/journal.pone.0135002)
Supplement: S1 File — (DOC) [file pone.0135002.s001.doc]

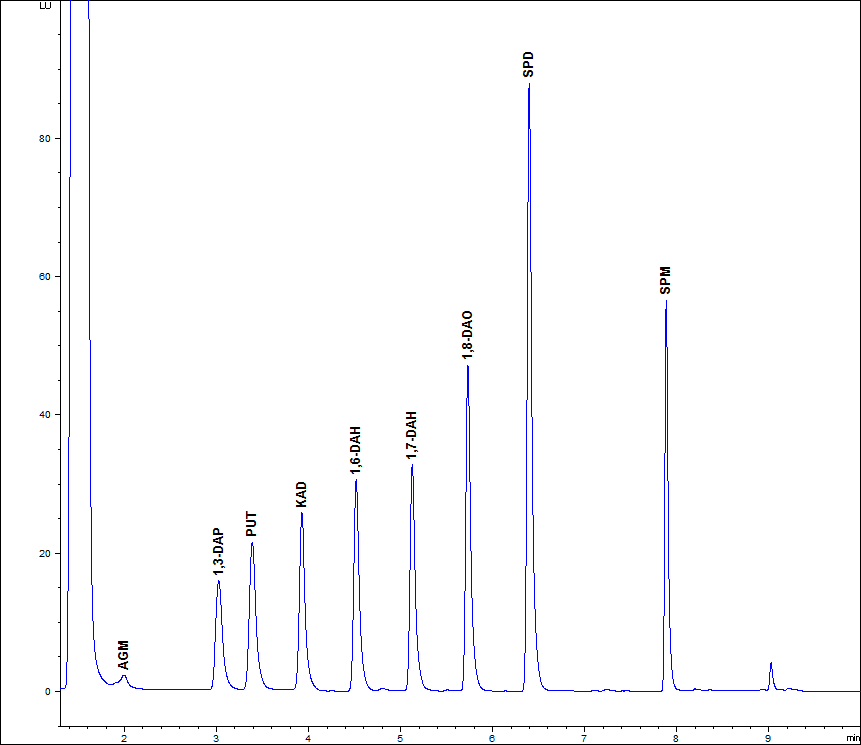


Chromatogram of polyamines pure standards mixture. AGM- agamatine, 1,3-DAP 1,3-diaminopentane, PUT- putrescine, KAD – kadaverine, 1,6-DAH 1,6-diaminohexane, 1,7-DAH 1,7-diamonoheptane, 1,8-DAO 1,8-diaminooctane, SPD – spermidine, SPM – spermine.


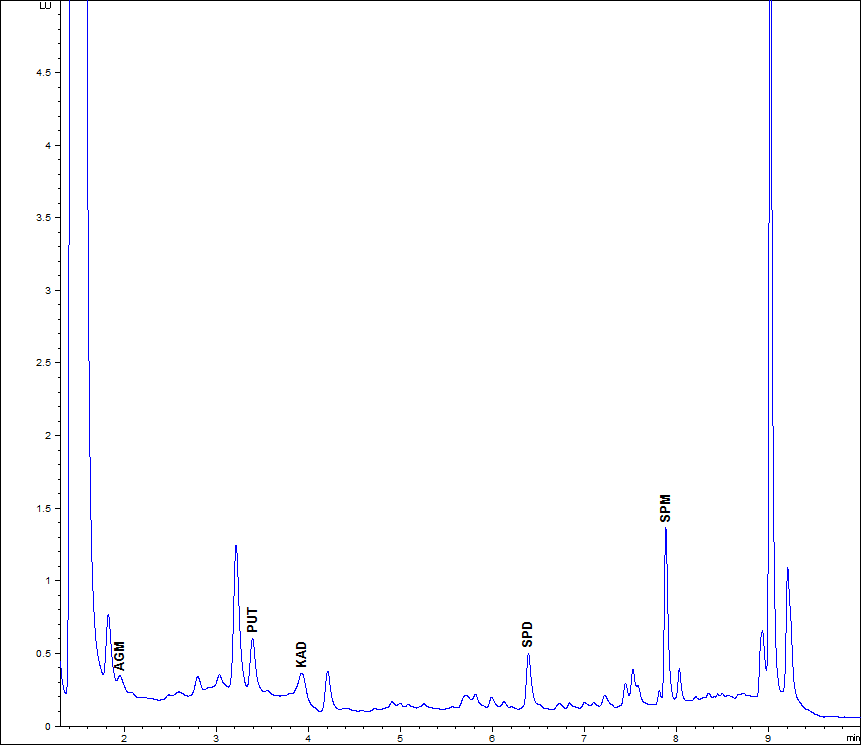


Drought treated Moderato typical chromatogram.


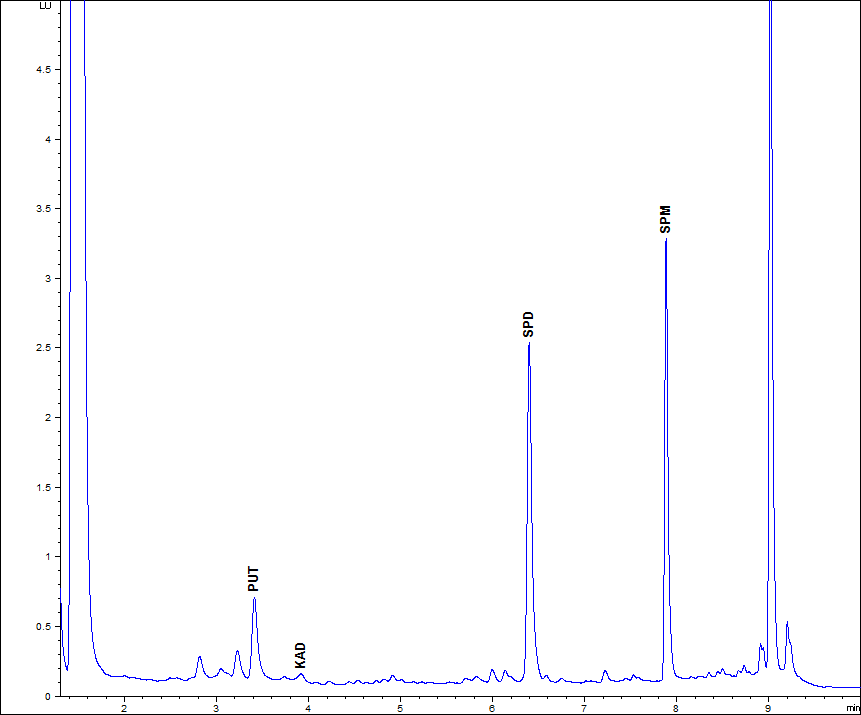


Moderato control conditions typical plant sample chromatogram.


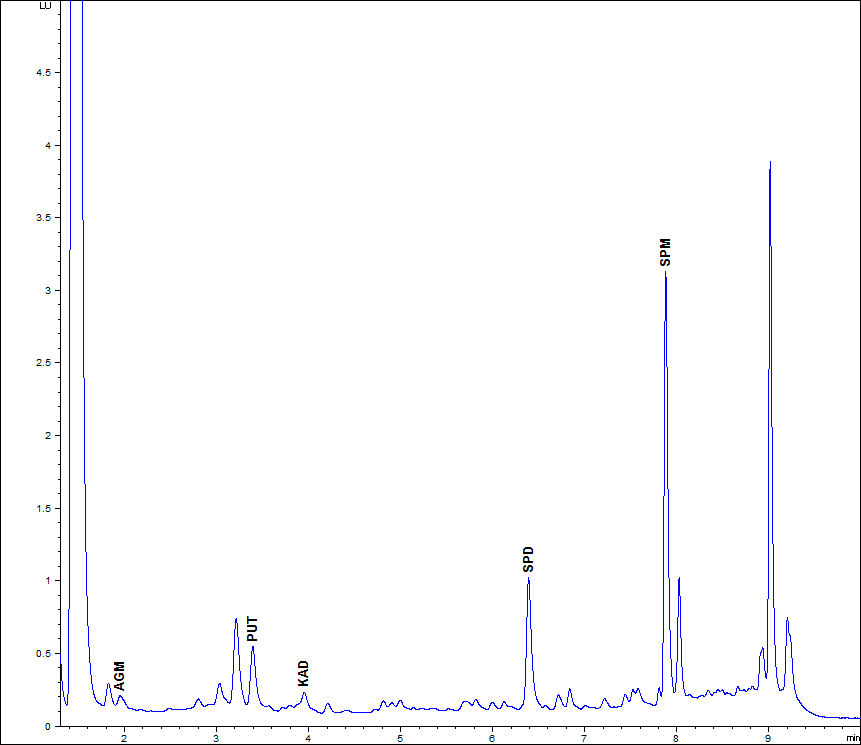


Drought treated Woltario typical chromatogram.


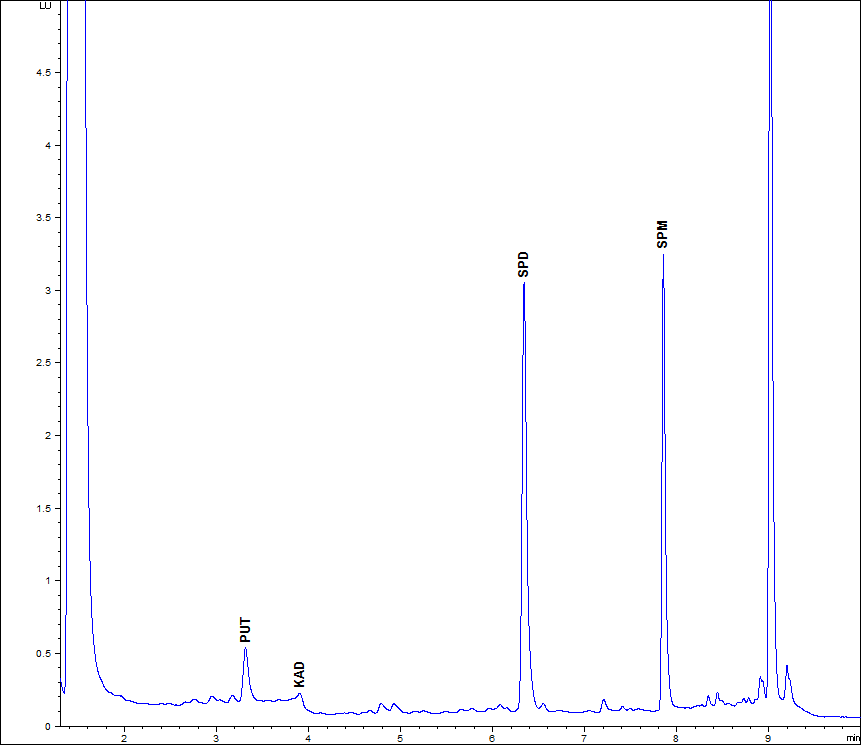


Woltario control conditions typical plant sample chromatogram.


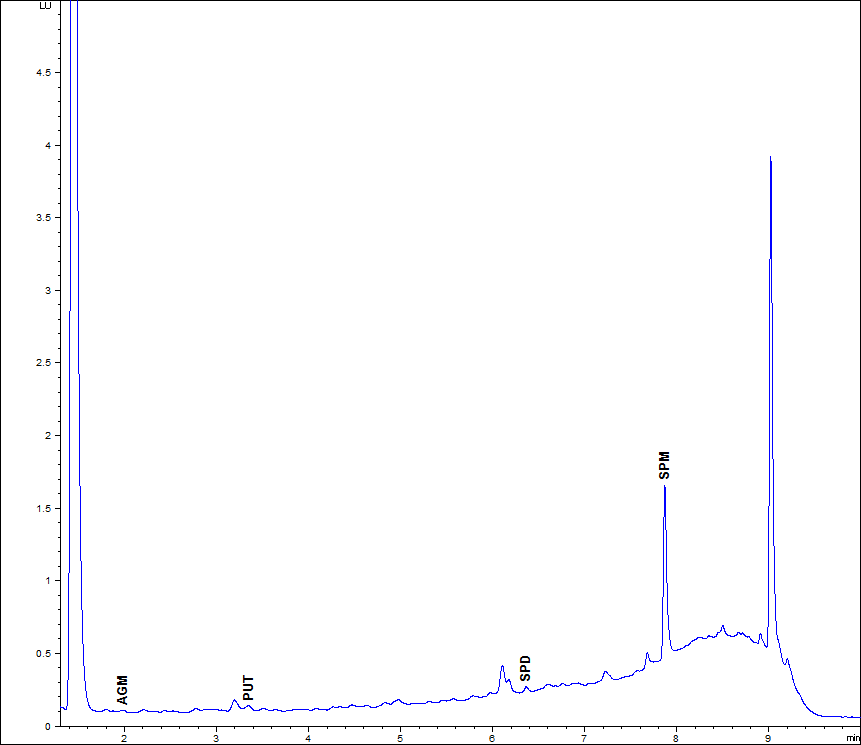


Drought treated Moderato typical chromatogram of cell wall bounded polyamines.


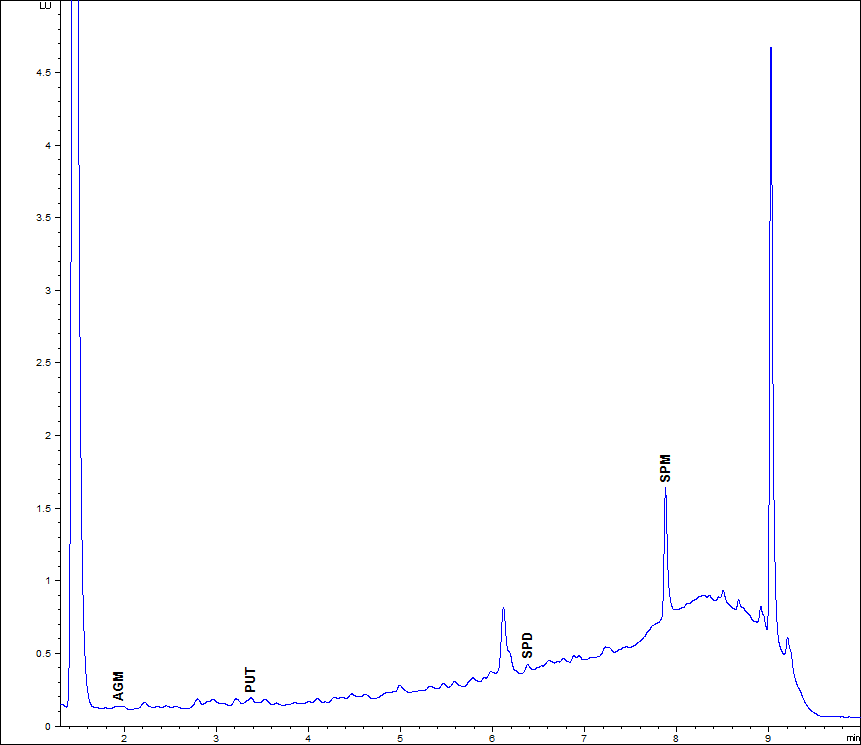


Moderato control conditions typical plant sample chromatogram of cell wall bounded polyamines.


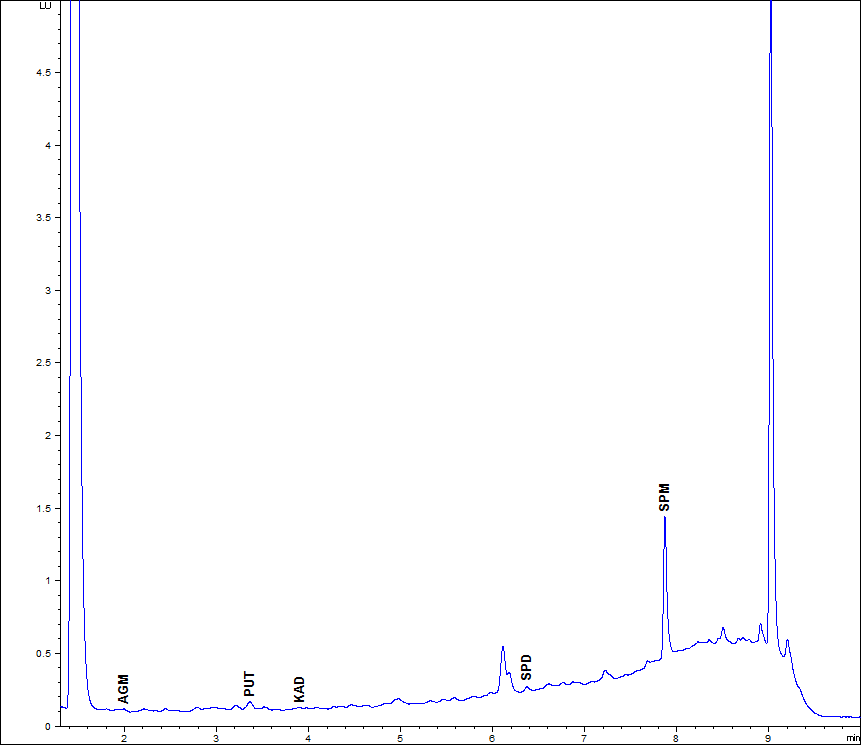


Drought treated Woltario typical chromatogram of cell wall bounded polyamines.


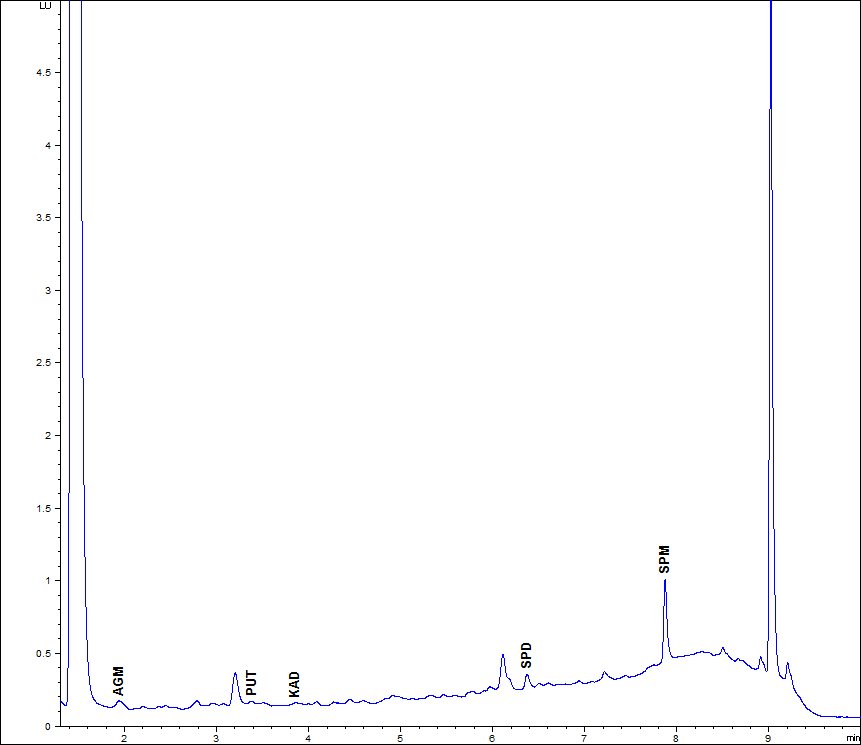


Woltario control conditions typical plant sample chromatogram of cell wall bounded polyamines.
